# Dataset for a *Dugesia japonica* de novo transcriptome assembly, utilized for defining the voltage-gated like ion channel superfamily

**DOI:** 10.1016/j.dib.2016.11.022

**Published:** 2016-11-16

**Authors:** John D. Chan, Dan Zhang, Xiaolong Liu, Magdalena Z. Zarowiecki, Matthew Berriman, Jonathan S. Marchant

**Affiliations:** a Department of Pharmacology, University of Minnesota Medical School, MN 55455, USA; b Parasite Genomics Group, Wellcome Trust Sanger Institute, Hinxton, United Kingdom; c The Stem Cell Institute, University of Minnesota Medical School, MN 55455, USA

## Abstract

This data article provides a transcriptomic resource for the free living planarian flatworm *Dugesia japonica* related to the research article entitled ‘Utilizing the planarian voltage-gated ion channel transcriptome to resolve a role for a Ca^2+^ channel in neuromuscular function and regeneration (J.D. Chan, D. Zhang, X. Liu, M. Zarowiecki, M. Berriman, J.S. Marchant, 2016) [1]. Data provided in this submission comprise sequence information for the unfiltered de novo assembly, the filtered assembly and a curated analysis of voltage-gated like (VGL) ion channel sequences mined from this resource. Availability of this data should facilitate further adoption of this model by laboratories interested in studying the role of individual genes of interest in planarian physiology and regenerative biology.

**Specifications Table**

**Table:** 

Subject area	*Biology*
More specific subject area	*Transcriptomics*
Type of data	*Sequence data (*2 *files), Summary Table referencing VGL information*
How data was acquired	*RNAseq, de novo assembly, post-hoc curation*
Data format	*Text files (.fasta). Unfiltered assembly (Dataset 1), Filtered assembly (Dataset 2). Table (.xls) of evidenced voltage-gated like ion channels* (Table 1)
Experimental factors	Sequencing was performed on samples from a clonal, asexual laboratory strain of the planarian *D. japonica* (GI strain) and this data used to generate a *de novo* transcriptome assembly.
Experimental features	The resulting assembly was analyzed for the presence of members of the voltage-gated like (VGL) superfamily of ion channels.
Data source location	*n/a*
Data accessibility	*Analyzed and filtered datasets are contained within this article.*

**Value of the data**

• Provision of a *de novo* transcriptome assembly for *Dugesia japonica* will act as a resource to facilitate investigation of the role of individual genes in this model system.
• Curation of the voltage-gated ion channel like superfamily in this system provides a benchmark for further annotation and study of the role of these channels in planarian regenerative physiology.

## 1 Data

The dataset of this article comprises three data files as follows: (i) Dataset 1. FASTA file (*raw_trinity_assembly.fasta*) of the unfiltered Trinity assembly, (ii) Dataset 2. FASTA file (*filtered_trinity_cds.fasta*) of the filtered Trinity assembly containing 44,857 contigs, and (iii) Table 1: voltage gated like (VGL) ion channel sequences resolved from the *Dugesia japonica* transcriptome. Contig IDs for ion channel sequences contained in the *D. japonica de novo* assembly organized by putative VGL ion channel family following manual inspection of transmembrane helix organization, structural motifs and ion selectivity residues. FPKM values reflect expression levels in whole (non-regenerating) animals. Additional analysis of these datasets are presented in the associated publication (‘Utilizing the planarian voltage-gated ion channel transcriptome to resolve a role for a Ca^2+^ channel in neuromuscular function and regeneration’, Chan et al. [1]).

## 2 Experimental design, materials and methods

Sequencing was performed on individuals from a clonal, asexual laboratory strain of the planarian *D. japonica* (GI strain). In order to sample a diversity of expressed transcripts, total RNA was extracted from intact (non-regenerating) worms (3 biological replicates of 100 individuals), as well as anterior worm fragments harvested at various intervals following tail amputation (1, 12, 24 h; 3 biological replicates of 200 heads per time point) using Trizol reagent. mRNA was purified using oligo(dT) beads (Dynal), yielding approximately 2 µg mRNA per biological sample. RNA-seq libraries were prepared according to the Illumina mRNA-Seq Sample Prep kit and Illumina TruSeq kit manufacturer protocols. Libraries were sequenced on Illumina HiSeq 2000 machines (Sanger Center, Hinxton) and the resulting 100 bp paired end reads were processed with Trimmomatic version 0.22 [2] to remove adapter sequences and low quality reads (sliding window quality filter, window size=4, minimum average quality score=25) while retaining reads ≥50 bp. In order to generate the *de novo* transcriptome assembly, overlapping paired-end reads were merged using FLASH [3] and fed into the Trinity pipeline [4], carried out with a minimum k-mer coverage of 2 and default k-mer size of 25. Graphs not resolving within a 6 h window were excised to allow the assembly to proceed and the minimum contig or transcript length was set to 100 nt. Relative transcript abundance was estimated using bowtie (version 2) to align trimmed reads to the *de novo* assembly and RSEM (version 1.2.11) to quantify read mapping, yielding FPKM (Fragments Per Kilobase of transcript per Million mapped reads) values for each contig. Assembled contigs were annotated using the TransDecoder package to predict translated open reading frames, which were searched against the NCBI Conserved Domain Database.

The initial Trinity *de novo* assembly of *D. japonica* RNAseq data produced a dataset with 195,271 sequences and an N50 of 1,587 bp. This number of contigs exceeds the number of predicted gene models in published flatworm genomes [5], [6], [7], [8], [9], likely due to a high number of redundant or incorrectly/partially assembled transcripts in the *D. japonica* assembly. Therefore, this preliminary dataset was filtered to retain only (i) sequences with predicted open reading frames (ORFs) ≥100 amino acids that contain an assignment to a known PFAM structural domain, or (ii) sequences with predicted open reading frames (ORFs) ≥100 amino acids that were evidenced by read mapping (FPKM value ≥1). The resulting filtered assembly retained 44,857 sequences with an N50 of 2444 bp. Sequences from the unfiltered assembly are provided as **Dataset 1.** The filtered ORF assembly of 44,857 sequences is provided **Dataset 2**.

Sequences belonging to the voltage-gated like ion channel superfamily were then curated by searching the translated *D. japonica* transcriptome for Pfam protein family hits corresponding to domains such as ion transport (PF00520, PF07885, PF08412), Ca_v_ (PF08763), Na_v_ (PF06512), PKD (PF08016), BK (PF03493), SK (PF035630) or cyclic nucleotide gated channels (PF08412, PF00027). Sequences were inspected to confirm the presence of the appropriate number of transmembrane helixes and pore forming domains and expected architecture/topology for each family of ion channels. This analysis resulted in the prediction of 114 unique pore-containing channel sequences that could be assigned to VGL ion channel families.

The appended Table 1 details the contig identifier and assignment of each of the *D. japonica* sequences based upon our assembly and current filtering methods. Within each class, assignments are ordered by FPKM values (fragments per kilobase of transcript per million mapped reads) to convey which transcripts predominate within each class of channels.
